# Supplementary material for: Interlaboratory Performance of a Real-Time PCR Method for Detection of Ceratocystis platani, the Agent of Canker Stain of Platanus spp
Source: J Fungi (Basel). 2022 Jul 26;8(8):778. doi: 10.3390/jof8080778 (PMC9330143; doi:10.3390/jof8080778)
Supplement: Supplementary file 1 [file jof-08-00778-s001.zip › Table_S1.pdf]

Title:

Interlaboratory performance of a Real-Time PCR method for detection of *Ceratocystis platani*, the agent of canker stain of *Platanus* spp.

Journal name:

Journal of Fungi

Authors:

Angela Brunetti, Kurt Heungens, Jacqueline Hubert, Renaud Ioos, Gian Luca Bianchi, Francesca De Amicis, Anne Chandelier, Sietse Van Der Linde, Ana Perez-Sierra, Valeria Gualandri, Maria Rosaria Silletti, Vito Nicola Trisciuzzi, Silvia Rimondi, Tiziana Baschieri, Elio Romano, Valentina Lumia, Marta Luigi, Francesco Faggioli, and Massimo Pilotti

Corresponding author:

Massimo Pilotti

Affiliation:

Institution: Council for Agricultural Research and Economics; Department: Research Center for Plant Protection and Certification (CREA-DC) - Via C. G. Bertero 22, 00156 Rome, Italy

E-mail address:

massimo.pilotti@crea.gov.it

Table S1 - The organizing and the participating laboratories (OL and PL) of the test performance study (TPS) for *Ceratocystis platani* in-wood detection. PL are listed alphabetically relating the surname of the first author from each laboratory.

Participants	Addresses
Angela Brunetti.<br>Valentina Lumia and<br>Massimo Pilotti<br>(OL)	Council for Agricultural Research and Economics<br>Research Centre for Plant Protection and Certification - CREA-DC<br>Rome (Italy), ZIP code 00156<br>angela.brunetti@crea.gov.it<br>valentina.lumia@crea.gov.it<br>massimo.pilotti@crea.gov.it
Gian Luca Bianchi,<br>Francesca De Amicis<br>(PL)	Agenzia regionale per lo sviluppo rurale - ERSA<br>Servizio fitosanitario e chimico, ricerca, sperimentazione ed assistenza tecnica<br>Struttura stabile laboratorio di fitopatologia e biotecnologie<br>Pozzuolo del Friuli, Udine (Italy) ZIP code 33050<br>gianluca.bianchi@ersa.fvg.it<br>francesca.deamicis@ersa.fvg.it
Anne Chandelier<br>(PL)	Walloon Agricultural Research Centre - CRA-W<br>Life Sciences Department<br>Gembloux (Belgium) ZIP code 5030<br>a.chandelier@cra.wallonie.be
Valeria Gualandri<br>(PL)	FEM-IASMA, Centro Trasferimento Tecnologico<br>Dipartimento Sperimentazione e Servizi Tecnologici<br>Unità Protezione Piante e Biodiversità Agroforestale<br>S. Michele all'Adige, Trento (Italy) ZIP code 38098<br>valeria.gualandri@fmach.it
Kurt Heungens<br>(PL)	Flanders Research Institute for Agriculture, Fisheries and Food - ILVO<br>Merelbeke (Belgium) ZIP code 9940<br>kurt.heungens@ilvo.vlaanderen.be
Jacqueline Hubert,<br>Renaud Ioos<br>(PL)	Plant Health Laboratory for the French Agency for Food, Environmental and Occupational Health & Safety (ANSES)<br>Mycology unit<br>Malzéville (France) ZIP code 54220<br>jacqueline.hubert@anses.fr<br>renaud.ioos@anses.fr
Silvia Rimondi<br>Tiziana Baschieri<br>(PL)	Servizio Fitosanitario Regione Emilia-Romagna<br>Bologna (Italy) ZIP code 40129<br>silvia.rimondi@regione.emilia-romagna.it<br>tiziana.baschieri@regione.emilia-romagna.it
Maria Rosaria Silletti<br>Vito Nicola Trisciuzzi<br>(PL)	Centro di Ricerca, Sperimentazione e Formazione in Agricoltura BASILE CARAMIA<br>Locorotondo, Bari (Italy) ZIP code 70010<br>mariarsilletti@crsfa.it<br>nik.tris@gmail.com
Sietse Van Der Linde<br>Ana Perez-Sierra<br>(PL)	Forest Research<br>Tree Health Diagnostic & Advisory Service<br>Alice Holt Lodge, Farnham Surrey (United Kingdom) ZIP code GU10 4LH<br>s.vanderlinde@nvwa.nl<br>ana.perez-sierra@forestresearch.gov.uk
