# Supplementary material for: Interlaboratory Performance of a Real-Time PCR Method for Detection of Ceratocystis platani, the Agent of Canker Stain of Platanus spp
Source: J Fungi (Basel). 2022 Jul 26;8(8):778. doi: 10.3390/jof8080778 (PMC9330143; doi:10.3390/jof8080778)
Supplement: Supplementary file 1 [file jof-08-00778-s001.zip › Table_S2.pdf]

Title:

Interlaboratory performance of a Real-Time PCR method for detection of *Ceratocystis platani*, the agent of canker stain of *Platanus* spp.

Journal name:

Journal of Fungi

Authors:

Angela Brunetti, Kurt Heungens, Jacqueline Hubert, Renaud Ioos, Gian Luca Bianchi, Francesca De Amicis, Anne Chandelier, Sietse Van Der Linde, Ana Perez-Sierra, Valeria Gualandri, Maria Rosaria Silletti, Vito Nicola Trisciuzzi, Silvia Rimondi, Tiziana Baschieri, Elio Romano, Valentina Lumia, Marta Luigi, Francesco Faggioli, and Massimo Pilotti

Corresponding author:

Massimo Pilotti

Affiliation:

Institution: Council for Agricultural Research and Economics; Department: Research Center for Plant Protection and Certification (CREA-DC) - Via C. G. Bertero 22, 00156 Rome, Italy

E-mail address:

massimo.pilotti@crea.gov.it

Table S2 - Scheduled activities of the organizing laboratory (OL = Z) and the participating laboratories (PL) within the test performance study (TPS) for *Ceratocystis platani* detection.

Activities<br><br>Participant laboratories (PL)	I step:<br>Establish the best annealing/extension time	II step:<br>The stability test	III step:<br>The standard curves (*sensu strictu* TPS)	IV step:<br>Testing unknown samples (*sensu strictu* TPS)	V step:<br>Analytical sensitivity	Real-Time PCR systems used
A	• Taqman with Eurogentec master mix	• Taqman with Bio-Rad master mix	• Taqman with Bio-Rad master mix<br>• Taqman with Eurogentec master mix	• Taqman with Bio-Rad master mix<br>• Taqman with Eurogentec master mix	• Taqman with Bio-Rad master mix<br>• Taqman with Eurogentec master mix	CFX96 (Bio-Rad)<br><br>StepOne Plus™ (Applied Biosystem, Thermo Fisher Scientific)
B	• Taqman with Thermo Scientific master mix	• Taqman with Bio-Rad master mix	• Taqman with Bio-Rad master mix<br>• Taqman with Thermo Scientific master mix<br>• SYBR Green with Thermo Scientific master mix	• Taqman with Bio-Rad master mix<br>• Taqman with Thermo Scientific master mix<br>• SYBR Green with Thermo Scientific master mix	• Taqman with Bio-Rad master mix	CFX96™ (Bio-Rad)
C	• Taqman with Eurogentec master mix	• Taqman with Bio-Rad master mix	• Taqman with Bio-Rad master mix<br>• Taqman with Eurogentec master mix	• Taqman with Bio-Rad master mix<br>• Taqman with Eurogentec master mix	• Taqman with Bio-Rad master mix	Rotor-Gene ™ 6000 (Corbett, Life Science)
D	N.P. [1]	• Taqman with Bio-Rad master mix<br>• EvaGreen with Bio-Rad master mix	• EvaGreen with Bio-Rad master mix	• Taqman with Bio-Rad master mix<br>• EvaGreen with Bio-Rad master mix	N.P.	Lightcycler 480 (Roche)
E	N.P.	• Taqman with Bio-Rad master mix<br>• EvaGreen with Bio-Rad master mix	• Taqman with Bio-Rad master mix<br>• EvaGreen with Bio-Rad master mix	• Taqman with Bio-Rad master mix<br>• EvaGreen with Bio-Rad master mix	• Taqman with Bio-Rad master mix<br>• EvaGreen with Bio-Rad master mix	CFX96™ (Bio-Rad)

Activities<br><br>Participant laboratories (PL)	I step:<br>Establish the best annealing/extension time	II step:<br>The stability test	III step:<br>The standard curves (*sensu strictu* TPS)	IV step:<br>Testing unknown samples (*sensu strictu* TPS)	V step:<br>Analytical sensitivity	Real-Time PCR systems used
F	N.P.	• Taqman with Bio-Rad master mix<br>• EvaGreen with Bio-Rad master mix	N.P.	• Taqman with Bio-Rad master mix<br>• EvaGreen with Bio-Rad master mix	• Taqman with Bio-Rad master mix	CFX96™ (Bio-Rad)
G	N.P.	• Taqman with Bio-Rad master mix<br>• EvaGreen with Bio-Rad master mix	• EvaGreen with Bio-Rad master mix	• Taqman with Bio-Rad master mix<br>• EvaGreen with Bio-Rad master mix	• Taqman with Bio-Rad master mix	CFX96™ (Bio-Rad)
H	• SYBR Green with Applied Biosystems master mix	• SYBR Green with Bio-Rad master mix	N.P.	• SYBR Green with Applied Biosystems master mix<br>• SYBR Green with Bio-Rad master mix	• SYBR Green with Applied Biosystems master mix<br>• SYBR Green with Bio-Rad master mix	Applied Biosystems® 7500 (Applied Biosystem, Thermo Fisher Scientific)
Z (OL)[2]	N.P.	N.P.	• Taqman with Bio-Rad supermix<br>• EvaGreen with Bio-Rad supermix<br>• SYBR Green with Bio-Rad supermix	• Taqman with Bio-Rad supermix<br>• EvaGreen with Bio-Rad supermix<br>• SYBR Green with Bio-Rad supermix	• Taqman with Bio-Rad supermix<br>• EvaGreen with Bio-Rad supermix<br>• SYBR Green with Bio-Rad supermix	CFX96™ (Bio-Rad)

[1] N.P. = Not Performed
[2]The activity reported for the OL regards its participation as a PL. See the text for the activity as a OL *sensu strictu*
