# Supplementary material for: Interlaboratory Performance of a Real-Time PCR Method for Detection of Ceratocystis platani, the Agent of Canker Stain of Platanus spp
Source: J Fungi (Basel). 2022 Jul 26;8(8):778. doi: 10.3390/jof8080778 (PMC9330143; doi:10.3390/jof8080778)
Supplement: Supplementary file 1 [file jof-08-00778-s001.zip › Table_S3.pdf]

Title:

Interlaboratory performance of a Real-Time PCR method for detection of *Ceratocystis platani*, the agent of canker stain of *Platanus* spp.

Journal name:

Journal of Fungi

Authors:

Angela Brunetti, Kurt Heungens, Jacqueline Hubert, Renaud Ioos, Gian Luca Bianchi, Francesca De Amicis, Anne Chandelier, Sietse Van Der Linde, Ana Perez-Sierra, Valeria Gualandri, Maria Rosaria Silletti, Vito Nicola Trisciuzzi, Silvia Rimondi, Tiziana Baschieri, Elio Romano, Valentina Lumia, Marta Luigi, Francesco Faggioli, and Massimo Pilotti

Corresponding author:

Massimo Pilotti

Affiliation:

Institution: Council for Agricultural Research and Economics; Department: Research Center for Plant Protection and Certification (CREA-DC) - Via C. G. Bertero 22, 00156 Rome, Italy

E-mail address:

massimo.pilotti@crea.gov.it

Table S3 - Detection of *Ceratocystis platani* with morphological/biological assays in the plane tree wood samples collected for the test performance study (TPS).

	Microscope assay	Moist chamber	Carrot assay	Isolation	Bait-plant assay
Naturally-*C.platani*-infected tree 1 (NI.1)	+	-	+	-	N.P.
Naturally-*C.platani*-infected tree 2 (NI.2)	+	-	+	+	N.P.
Naturally-*C.platani*-infected tree 3 (NI.3)	+	+	+	+	N.P.
Artificially-*C.platani*-infected tree 1 (AI.1)	-	-	-	-	+
Artificially-*C.platani*-infected tree 2 (AI.2)	-	-	-	-	+
Artificially-*C.platani*-infected tree 3 (AI.3)	-	-	-	-	+
Artificially-*C.platani*-infected tree 4 (AI.4)	-	-	-	-	+
Artificially-*C.platani*-infected tree 5 (AI.5)	-	-	-	-	+
Artificially-*C.platani*-infected tree 6 (AI.6)	-	-	-	-	+
Healthy tree 1 (H.1)	-	-	-	-	N.P.
Healthy tree 2 (H.2)	-	-	-	-	N.P.
Healthy tree 3 (H.3)	-	-	-	-	N.P.
Diseased tree 1 (D.1) (*C. platani*-non-infected)	-	-	-	-	-
Diseased tree 2 (D.2) (*C. platani*-non-infected)	-	-	-	-	-
Diseased tree 3 (D.3) (*C. platani*-non-infected)	-	-	-	-	-

N.P. = Not Performed
