# Supplementary material for: Interlaboratory Performance of a Real-Time PCR Method for Detection of Ceratocystis platani, the Agent of Canker Stain of Platanus spp
Source: J Fungi (Basel). 2022 Jul 26;8(8):778. doi: 10.3390/jof8080778 (PMC9330143; doi:10.3390/jof8080778)
Supplement: Supplementary file 1 [file jof-08-00778-s001.zip › Text_S1.pdf]

Title:

Interlaboratory performance of a Real-Time PCR method for detection of *Ceratocystis platani*, the agent of canker stain of *Platanus* spp.

Journal name:

Journal of Fungi

Authors:

Angela Brunetti, Kurt Heungens, Jacqueline Hubert, Renaud Ioos, Gian Luca Bianchi, Francesca De Amicis, Anne Chandelier, Sietse Van Der Linde, Ana Perez-Sierra, Valeria Gualandri, Maria Rosaria Silletti, Vito Nicola Trisciuzzi, Silvia Rimondi, Tiziana Baschieri, Elio Romano, Valentina Lumia, Marta Luigi, Francesco Faggioli, and Massimo Pilotti

Corresponding author:

Massimo Pilotti

Affiliation:

Institution: Council for Agricultural Research and Economics; Department: Research Center for Plant Protection and Certification (CREA-DC) - Via C. G. Bertero 22, 00156 Rome, Italy

E-mail address:

massimo.pilotti@crea.gov.it

Text S1 - Biological assays used along with Real-Time PCR to assess the presence of *Ceratocystis platani* in the samples used for the test performance study (TPS).

Microscope assay. Thin slices of wood were cut with a razor blade in order to obtain the longitudinal section of the xylem vessels. The sections were observed under a microscope in order to assess the presence/absence of the aleurioconidia typical of *C. platani*.

Isolation on nutritive medium. Small wood fragments were rapidly flamed and plated on PDA (PDA, Oxoid-Unipath Ltd, Basingstoke, Hampshire, England) supplemented with 0.3gr/l streptomycin. We plated 36 fragments for each sample. The PDA plates were incubated at 20-25°C and observed for the emergence of the typical *C. platani* colonies for about ten days.

Carrot assay. Carrot slices were placed in glassy Petri plates 12 cm in diameter, a small wood fragment was inserted in the center of each slice; the slices were incubated at 20-25°C and periodically observed for development of the typical *C. platani* perithecia and mycelium for about ten days; each sample was tested with 15 carrot slices (five for each plate).

Moist chamber. Wood pieces were placed in Petri plates 12 cm in diameter which in turn were placed in Petri plates 19 cm in diameter flooded with water. Incubation was as described above, and the observations were aimed to find the typical *C. platani* perithecia in about fifteen days.

Bait-plant assay. One-year-old seedlings of *P.* × *acerifolia* were inoculated at the base of the stem by sterilizing with ethanol the bark surface in the inoculation point, cutting the bark top-down with a razor blade through the cambium and peripheral wood rings and inserting a thin splinter of wood 4 × 3 mm sized obtained from the wood of the trees to be diagnosed. The bark strip was then gently set on the splinter and the inoculation point was wrapped with parafilm. Three seedlings were inoculated for each sample-tree. Plants were observed for three months. In case canker stain symptoms appeared the presence of *C. platani* was confirmed by isolating on nutritive medium as above described.

The specific nature of perithecia and mycelium obtained in the various assays was ascertained by microscopic observations which enables the identification of C. platani conidia and ascospores (OEPP/EPPO 2014a).
