# Supplementary material for: Interlaboratory Performance of a Real-Time PCR Method for Detection of Ceratocystis platani, the Agent of Canker Stain of Platanus spp
Source: J Fungi (Basel). 2022 Jul 26;8(8):778. doi: 10.3390/jof8080778 (PMC9330143; doi:10.3390/jof8080778)
Supplement: Supplementary file 1 [file jof-08-00778-s001.zip › Text_S2.pdf]

Title:

Interlaboratory performance of a Real-Time PCR method for detection of *Ceratocystis platani*, the agent of canker stain of *Platanus* spp.

Journal name:

Journal of Fungi

Authors:

Angela Brunetti, Kurt Heungens, Jacqueline Hubert, Renaud Ioos, Gian Luca Bianchi, Francesca De Amicis, Anne Chandelier, Sietse Van Der Linde, Ana Perez-Sierra, Valeria Gualandri, Maria Rosaria Silletti, Vito Nicola Trisciuzzi, Silvia Rimondi, Tiziana Baschieri, Elio Romano, Valentina Lumia, Marta Luigi, Francesco Faggioli, and Massimo Pilotti

Corresponding author:

Massimo Pilotti

Affiliation:

Institution: Council for Agricultural Research and Economics; Department: Research Center for Plant Protection and Certification (CREA-DC) - Via C. G. Bertero 22, 00156 Rome, Italy

E-mail address:

massimo.pilotti@crea.gov.it

Text S2 - Information on fluorophores and the Real-Time PCR systems used in the test performance study (TPS) for *Ceratocystis platani* detection.

Fluorophores

EvaGreen, SYBR Green I, 6-FAM (6-carboxy-fluorescein), were the fluorophores used. In presence of DNA EvaGreen has a maximum absorbance at 500 nm with a shoulder peak at 470 nm, and a maximum emission at 530 nm [36]. SYBR Green I has a maximum absorbance at 497 nm, with secondary excitation peaks roughly at ~ 290 and ~ 380. Maximum emission is at 520 nm (The Molecular Probes Handbook, Thermo Fisher Scientific, https://www.thermofisher.com/it/en/home/references/molecular-probes-the-handbook/nucleic-acid-detection-and-genomics-technology.html). The absorbance and emission spectrum of FAM is pH-sensitive. Maximum absorbance is at pH 8-9, and 494-498 nm. Maximum emission is also at these pH values and at 517-518 nm. [37] (see also: The Molecular Probes Handbook, Thermo Fisher Scientific, https://www.thermofisher.com/it/en/home/references/molecular-probes-the-handbook/fluorophores-and-their-amine-reactive-derivatives.html)

Real-Time PCR systems

Here below we report the Real-Time PCR systems used and the excitation and detection sources set up for the fluorophores.

1. CFX96™ (Bio-Rad): for all three fluorophores, the dedicated excitation led was channel 1 (450-490 nm) and the photo-body detector was channel 1 (515-530nm).
2. StepOne Plus™ (Applied Biosystem, Thermo Fisher Scientific): the excitation source for FAM was at 470 nm and the detection source was at 520 nm.
3. Rotor-Gene ™ 6000 (Corbett, Life Science): the excitation source for FAM was at 470 nm and the detection source was at 510 nm.
4. Lightcycler 480 (Roche): for EvaGreen and FAM the excitation source was at 465 nm and the detection at 510 nm.
5. Applied Biosystems® 7500 (Applied Biosystem, Thermo Fisher Scientific): Informations not found

In all Real-Time PCR systems threshold line was set in an auto-calculating modality.

Citations

36. Mao, F.; Leung, W.Y.; Xin, X. Characterization of EvaGreen and the implication of its physicochemical properties for qPCR applications. *BMC Biotechnol.* **2007**, *7*, 76. doi: 10.1186/1472-6750-7-76

37. Sjöback, R.; Nygren, J.; Kubista, M. Absorption and Fluorescence Properties of Fluorescein. *Spectrochim. Acta A-M*, **1995**, *51*(6), L7-L21. https://doi.org/10.1016/0584-8539(95)01421-P
