# Supplementary material for: Interlaboratory Performance of a Real-Time PCR Method for Detection of Ceratocystis platani, the Agent of Canker Stain of Platanus spp
Source: J Fungi (Basel). 2022 Jul 26;8(8):778. doi: 10.3390/jof8080778 (PMC9330143; doi:10.3390/jof8080778)
Supplement: Supplementary file 1 [file jof-08-00778-s001.zip › Text_Table_S1.pdf]

Title:

Interlaboratory performance of a Real-Time PCR method for detection of *Ceratocystis platani*, the agent of canker stain of *Platanus* spp.

Journal name:

Journal of Fungi

Authors:

Angela Brunetti, Kurt Heungens, Jacqueline Hubert, Renaud Ioos, Gian Luca Bianchi, Francesca De Amicis, Anne Chandelier, Sietse Van Der Linde, Ana Perez-Sierra, Valeria Gualandri, Maria Rosaria Silletti, Vito Nicola Trisciuzzi, Silvia Rimondi, Tiziana Baschieri, Elio Romano, Valentina Lumia, Marta Luigi, Francesco Faggioli, and Massimo Pilotti

Corresponding author:

Massimo Pilotti

Affiliation:

Institution: Council for Agricultural Research and Economics; Department: Research Center for Plant Protection and Certification (CREA-DC) - Via C. G. Bertero 22, 00156 Rome, Italy

E-mail address:

massimo.pilotti@crea.gov.it

Text/Table S1 - Basic information and conditions of the Real-Time PCR method for *Ceratocystis platani* detection used in the test performance study (TPS) by the organizing laboratory (OL) and the participating laboratories (PL): i) primers and probe sequences, ii) composition of master mixes, iii) reaction assembly, iv) thermal cycling conditions, v) establishing the best annealing time of Real-Time PCR based on master mixes other than Bio-Rad.

Primers and probe sequences

The PCR target was the Internal Transcribed Spacer 1 region (ITS1) 95bp long

- Forward primer: C.P.Sn.For.I 5'-CGTACCTATCTTGTAGTGAGATGAATGC-3' (from position 89 to 116 of the *C. platani* reference sequence DQ399853).
- Reverse primer: C.P.Sn.Rev.I 5'-GAGTTTACAGTGGCGAGACTATACTG-3' (from position 158 to 183).
- Taqman probe: C.P.TM.Pr. 5'-CGGTGCCCTTCAGAAGGGCCCTACCACC-3', (from position 123 to 150). The probe was labeled with FAM (6- carboxy-fluorescein) at 5’ end, and contained Black Hole Quencher™ 1 (BHQ-1) at 3’ end.

List of master mixes used in the TPS:

BIO-RAD master mixes

- SsoFast™ EvaGreen® Supermix (for EvaGreen assay)
- SsoAdvanced™ Universal Probes Supermix (for Taqman assay)
- SsoAdvanced™ Universal SYBR® Green Supermix (for SYBR Green assay)

All three master mixes contain:

dNTPs, antibody-mediated hot-start Sso7d-fusion polymerase, $MgCl_2$, and stabilizers. In addition a blend of passive reference dyes, including ROX and fluorescein, is contained in the supermix for use in Taqman assay. The first and the third supermix contain respectively EvaGreen and SYBR Green I as the fluorescent dye

Master mixes from other companies

- qPCR MasterMix (Eurogentec, Seraing, Belgium). For Taqman assay.
  It contains: 2x reaction buffer contaning MgCl2, dNTP Mix, HotGoldStar DNA Polymerase, Uracyl-N-glycosilase, stabilizers and passive reference.

- qPCR MasterMix No ROX (Eurogentec, Seraing, Belgium). For Taqman assay.
  It contains: reaction buffer contaning dNTPs (including dUTP), HotGoldStar DNA Polymerase, MgCl2 (final concentration 5 mM), Uracil-N-Glycosilase, stabilizers.

- Maxima Probe/ROX qPCR Master Mix (Thermo Fisher Scientific, Waltham, Massachusetts). For Taqman assay.
  It contains: Maxima® Hot Start Taq DNA polymerase, dNTPs (including dUTP), Uracil-N-Glycosilase, KCl, (NH4)2SO4, and a ROX Passive reference dye.

- Maxima SYBR Green/ROX qPCR Master Mix. For SYBR Green assay.
  It contains: Maxima® Hot Start Taq DNA polymerase, dNTPs (including dUTP), Uracil-N-Glycosilase, KCl, (NH4)2SO4, SYBR Green I as the fluorescent intercalating dye, and a ROX Passive reference dye.

- Power SYBR Green PCR Master Mix (Applied Biosystem, Thermo Fisher Scientific, Waltham, Massachusetts). For SYBR Green assay.
  It contains: SYBR ® Green I Dye, AmpliTaq Gold® DNA Polymerase - UP, dNTPs, Passive reference, Optimized buffer components.

Reaction assembly

Reaction assembly with EvaGreen and SYBR Green dyes (any master mix)

Reagent	Working concentration	Volume per reaction (µl)	Final concentration
Molecular grade water *	N.A.	6	N.A.
Supermix	2X	10	1x
Forward Primer (C.P.Sn.For.I)	10 µM	1	0.5 µM
Reverse Primer (C.P.Sn.Rev.I)	10µM	1	0.5 µM
Subtotal		18	
DNA sample		2	
Total		20	

*Molecular grade and nuclease-free water should be preferably used.

Reaction assembly with Taqman probe (any master mix)

Reagent	Working concentration	Volume per reaction (µl)	Final concentration
Molecular grade water *	N.A.	5.7	N.A.
Supermix	2X	10	1x
Forward Primer (C.P.Sn.For.I)	10 µM	1	0.5 µM
Reverse Primer (C.P.Sn.Rev.I)	10 µM	1	0.5 µM
Probe (C.P.TM.Pr.)	20 µM	0.3	0.3 µM
Subtotal		18	
DNA sample		2	
Total		20	

* Molecular grade and nuclease-free water should be preferably used.

Thermal cycling conditions

In bold and grey-shaded are the changes made accordingly to the canonical thermal cycling conditions.

Bio-Rad master mixes in CFX96

Initial denaturation at 96°C for 3 min; 40 cycles at 95°C for 10 s (denaturation) and 66°C for 20 s (annealing/extension); final extension (for EvaGreen only) at 72 °C for 5 min. Only for EvaGreen and SYBR Green: dissociation run: increase of 0.5 °C, from 55 to 95 °C.

Bio-Rad supermixes in Rotor-Gene™ 6000 and Roche Lightcycler 480

Initial denaturation at 96°C for 3 min; 40 cycles at 95°C for 10 s (denaturation) and 66°C for **30 s** (annealing/extension).

Eurogentec, Thermo Scientific and Applied Biosystem supermixes

Initial denaturation at **95°C for 10 min**; 40 cycles at **95°C for 15 s** (denaturation) and 66°C for **a time to be experimentally determined**, (depending on the results of the preliminary test here below) (annealing/extension); final extension (for intercalating dye only) at 72 °C for 5 min. Only for SYBR Green: dissociation run: increase of 0.5 °C, from 55 to 95 °C.

Establish the best annealing time of Real-Time PCR based on master mixes other than Bio-Rad

Results of testing different annealing time at different concentrations

Chemistry<br>Master mix<br>(Company) (PL)	DNA titer	Average Ct value (standard deviation) at each annealing time: 20s	30s	40s	60s	Chosen value
Taqman<br>qPCR MasterMix<br>(Eurogentec) (A)	undiluted	19.7 (0.10)			16.4 (0.10)	**60s**
	1:1000	27.1 (0.09)			26.1 (0.15)	
Taqman<br>Maxima Probe/ROX qPCR Master Mix<br>(Thermo Scientific) (B)	undiluted	18.4 (0.20)	17.8 (0.18)	18.1 (0.18)		**30s**
	1:1000	28.7 (0.22)	28.0 (0.11)	28.3 (0.21)		
Taqman<br>qPCR MasterMix No ROX<br>(Eurogentec) (C)	undiluted	14.8 (0.05)			13.6 (0.16)	**60s**
	1:1000	23.8 (0.08)			23.5 (0.13)	
SYBR Green<br>Power SYBR Green PCR Master Mix<br>(Applied Biosystem) (H)	undiluted		15.9 (0.06)		15.9 (0.06)	**30s**
	1:1000		26 (0.06)		26 (0.18)	
